# Anxiety and depression disorders in oncological patients under palliative care at a hospital service: a cross-sectional study

**DOI:** 10.1186/s12904-023-01233-1

**Published:** 2023-08-15

**Authors:** Gustavo Souza Gontijo Garcia, Karina Cardoso Meira, Alessandra Hubner de Souza, Nathalia Sernizon Guimarães

**Affiliations:** 1 Faculdade Ciências Médicas de Minas Gerais, Belo Horizonte, Minas Gerais Brazil; 2 Escola de Saúde, Universidade Federal do Rio Grande do Norte, Natal, Rio Grande do Norte Brazil; 3 OPENS: Observatory of Epidemiology, Nutrition and Health Research, Faculdade Ciências Médicas de Minas Gerais/FELUMA, Belo Horizonte, Minas Gerais Brazil

**Keywords:** Cancer, Depression, Anxiety, Palliative care, Symptoms

## Abstract

**Background:**

This study aimed to evaluate the risk and protective factors associated with anxiety and depression symptoms in cancer patients at an advanced stage of cancer.

**Methods:**

A cross-sectional study was conducted on patients with advanced cancer who were receiving palliative care. Cancer patients aged 18 years or older, with preserved cognitive function who completed the questionnaires were eligible. The questionnaires of Hospital Anxiety and Depression Scale (HADS) and health related of quality of life questionnaire; the European Organization for Research and Treatment of Cancer (EORTC-C30) were applied. Outcome variables were the depression and anxiety symptoms of cancer patients under palliative care, according to the answers to the 14 items that make up the HADS Scale. The analysis used the R software, version 4.2.0.

**Results:**

Seventy cancer patients with advanced cancer were included. The colon was the most common neoplastic diagnostic (20%), followed by breast (12.9%) and lung (10%). The prevalence of depression was 44.3%, 25.7% anxiety and 52.9% had both symptoms. Patients with a high level of functionality had a lower chance of anxiety (OR = 0.80;p = 0.025), depression (OR = 0.82; p = 0.007), and anxiety and depression (OR = 0.82p = 0.008). We observed a lower chance of depression and depression/anxiety who showed a high level of Overall Performance. Three symptoms increased the chance of depression/anxiety: nausea/vomiting (p = 0.019), fatigue (0.031), loss of appetite (0.048).

**Conclusion:**

This study found high prevalence of anxiety and depression.Better quality of life and functionality were negatively associated with these outcomes. Examining the patient’s functions will assist the clinician in alleviating symptoms of anxiety and depression, giving cancer patients in palliative care more dignity.

**Trial registration:**

Not applicable.

## Introduction

Cancer is defined as a chronic disease characterized by irregular and disorderly cell development, which may invade adjacent tissues or distant organs, a process known as metastasis. The ability of tumor cells to multiply and invade different parts of the body distinguishes this disease [1, 2]. In the last century, we have observed a progressive increase in this disease due to population aging associated with changes in habits and lifestyle, such as the high prevalence of smoking, alcohol abuse, unhealthy diet, and sedentary lifestyle, among other factors [3]. In 2020, excluding non-melanoma skin cancer, 18.1 million new cases of cancer and 9.9 million deaths were estimated, and the World Health Organization estimates that annually more than 25.7 million people will need palliative care at the end of life in the world and that 28.5% of these are due to advanced cancer [4].

Cancer patients with advanced disease experience a significant burden of symptoms directly related to the cancer disease, its treatment (chemotherapy, radiotherapy, and surgery), treatment of symptoms, and pre-existing comorbidities [4–7]. The main symptoms experienced by cancer patients who need palliative care are pain, anxiety, depression, bleeding, confusion/delirium, constipation, dementia, depression, fatigue, shortness of breath, dry mouth, depression, diarrhea, shortness of breath, weakness, and wounds [4]. A systematic review study with meta-analysis pointed out that the most prevalent symptoms in these patients were fatigue (77.8%), urinary incontinence (77.5%), asthenia (66.7%), pain (66.3%), constipation (52.5%) and anxiety (50.0%) [5], it is noteworthy that patients may have more than one of these symptoms, and the multiplicity of symptoms will have an impact on their quality of life, increasing their mental suffering [8].

Depression and anxiety are the main symptoms of mental suffering experienced by patients with advanced cancer due to fear of death, loss of functional capacity for daily life activities, and intensification of symptoms due to the extension of the disease [9]. A systematic review with meta-analysis showed that the prevalence of depression in cancer patients undergoing palliative care was 27%; the highest magnitude was observed in the African continent (36%) and lowest in Europe (25%) [10]. The prevalence of anxiety in these patients shows great variability between studies, with values between 29% and 52% [7, 11, 12]. The frequency of these symptoms differs between types of cancer, in older individuals, with worse symptom control, lack of family support, and who showed the worse overall quality of life, lower level of physical, emotional, and cognitive functionality [8–[9, 11]–12].

Patients with cancer and comorbid depression have higher anxiety, pain, fatigue, and functioning than other cancer patients, as well as a greater risk of suicidal ideation and difficulty adhering to cancer treatments [13]. Moreover, they reveal more anxiety and depression symptoms than those with other chronic diseases, demonstrating the importance of detecting symptoms as soon as possible in order to provide a better life condition [14].

Integrating specific oncological treatments, such as chemotherapy, radiotherapy, or surgery, with palliative care improves symptom management and patient quality of life [15]. According to international recommendations and guidelines, people diagnosed with advanced cancer should receive early palliative care and active treatment simultaneously. To meet these guidelines palliative care is provided in most clinical practices by a multidisciplinary team that includes a palliative medicine specialist [16]. Given this context, cancer patients in palliative care must be frequently screened for depression and anxiety to identify associated factors and to improve clinical support and quality of life for patients with advanced and end-of-life diseases. That said, the present study aims to assess the prevalence of depression and anxiety in cancer patients undergoing palliative care and their association with sociodemographic and clinical variables and quality of life.

## Methods

### Design, sample, place and period of study

This is a prospective cross-sectional study involving patients with advanced cancer who are receiving palliative care. The research was carried out at Hospital Ibiapaba’s Oncology Sector in Barbacena, Minas Gerais, Brazil.

78 patients were invited and 70 were accepted to participate in the study, for a loss of 10.2% due to refusal to participate. The final sample included 70 cancer patients. Cancer patients in a palliative care outpatient clinic, aged 18 years or older, with preserved cognitive function, literate and semi-literate who completed the questionnaires were eligible. Patients with central nervous system metastases were excluded because they were unable to complete the questionnaire due to impaired cognition. Data was collected between October 2020 and April 2021.

### Data collection procedures and instruments

The research team was composed of the responsible researcher registered in Plataforma Brasil. The collection procedure was standardized, and the applicators were previously trained to guarantee the impartiality and reliability of the data to avoid information bias.

The research participants were contacted during routine consultations in which the reason for conducting the study and the objectives and procedures for data collection were explained. After checking whether they met the inclusion criteria and agreed to participate in the study, they had a face-to-face interview scheduled in a private location. They answered the questionnaire, which had three sections: sociodemographic variables (gender and age) clinical variables (tumor topography), in addition to two questionnaires.

Two questionnaires were used: (i) depression and anxiety scale in patients under hospital care (HADS scale) were validated for the Portuguese language; questionnaire; the European Organization for Research and Treatment of Cancer (EORTC-C30) and assessment of patient’s symptoms [17, 18].

Outcome variables were the depression and anxiety symptoms of cancer patients under palliative care, according to the answers to the 14 items that make up the HADS Scale. The questionnaire comprises seven questions for anxiety and seven questions for depression. Cut-off points correspond to mild between 0 and 7,moderate scores between 8 and 14 andsevere cases receive scores between 15 and 21. Patients with HADS ≥ 8 were considered to have depression or anxiety [18].

The European Organization for Research and Treatment of Cancer (EORTC) – EORTC QLQ-C30, version 3.0 – was used in this study as a dependent variable [17]. The questionnaire has thirty questions divided into multiple-item and single-item scales. The multi-item scales are Global Health/Quality of Life (QoL); Functional Scale, which is subdivided into five scales: physical function, role performance, emotional function, cognitive function, and social function; Symptom Scale, with symptoms of fatigue, pain, nausea and vomiting, and six unique items. For the first twenty-eight questions, the answers vary between nothing (1), little (2), moderate (3), or a lot (4). Questions about the general quality of life were answered on a scale of 1 to 7 (7 = excellent). Items generate a score ranging from 0 to 100. A high score for the Global Health/QoL and functional scale represents a high quality of life and functionality, respectively. A high score indicates a high level of symptoms/problems for the symptom scale. Responses were analyzed according to the EORTC QLQ-C30 scoring manual [20].

All instruments were validated for use in the Brazilian population, and showed high validity and reliability.The questionnaire data were entered into an electronic spreadsheet and analyzed using the R software, version 4.2.0 (R: A Language and Environment) for Statistical Computing, Vienna, Austria).

### Statistical analysis

Statistical analyses were performed in three main steps: (i) descriptive, (ii) univariate (evaluation of the association of the outcome with each variable of interest), and (iii) multivariate analyses.

In descriptive analyses, the categorical variables were summarized by absolute and relative frequency, and quantitative variables were summarized using median, and interquartile range (IQR). logistic regression models were used to calculate the unadjusted odds ratios and adjusted for sex, age, and by domains of the Quality of Life questionnaire of the European Organization for Research and Treatment of Cancer (EORTC-30), with the results of depression (yes/no) and anxiety symptoms (yes/no) and both conditions (yes/no). The scores for each questionnaire domain were entered into the model so that the estimated odds ratio represents the change in the outcome odds associated with a 10-unit increase in the questionnaire score. Subsequently, variables with p-values < 0.20 in the univariate analysis were simultaneously included in logistic regression models for each of the outcomes, were considered statistically significant p-values < 0,05. The statistical significance of the variables that were part of the models was evaluated by analyzing the OR and their respective 95% confidence intervals (95% CI), as well as by the p-value of the tests, aimed at reducing the probability of type I error. A comparison of the models’ goodness-of-fit tests was performed using the Akaike Information Criterion (AIC). The final multivariate model contained only the variables with 5% level of significance after adjusting for the other variables added to the previous multivariate models. The variables were included from the largest to the least significance, to test which associations between the explicative variables and the outcomes would remain significant throughout the process (p-value < 0.05) [19].All analyses were performed in R software version 4.0.2 0 (R: A Language and Environment) for Statistical Computing, Vienna, Austria).

### Ethics

The study was conducted in accordance with the Declaration of Helsinki (as revised in 2013). The project was approved by the research ethics committee of the Medicine Faculty of Barbacena under code CAAE: 33649720.2.0000.8307. All methods were performed in accordance with the relevant guidelines and regulations. Consent to participate in these studies was obtained from participants.

## Results

From 78 volunteers, 70 cancer patients diagnosed with advanced stage were included. From them, the majority (60%) were female, the median age was 60 (IQR = 58.2–74.0). The predominance of more aggressive tumors (58.6%). The most frequent neoplastic topography in the sample was colon (20%), followed by breast cancer (12.9%), lung (/10%), rectum (/10%), stomach (/7.1%), pancreas (7.1%), esophagus (5.7%), unknown (/4, 3%), ovary (4.3%), prostate (4.3%), melanoma (2.9%), nasopharynx (2.9%), bladder (1.4%), larynx (1.4%), CML (1.4%), oropharynx (1.4%), penis (1.4%) and kidney (n = 1/1.4%).

The tumor type was classified into: aggressive and less aggressive tumors. We considered more aggressive (rectum, pancreas and kidney) (7.31%) and less aggressive (prostate and breast) (6.9%) tumors considering the natural history of the disease, which led to the classification of more aggressive and less aggressive.According to the HADS questionnaire, the prevalence of depression was 44.3% (95%CI 32.7 to 55.9%), 25.7% (95%CI 15.5 to 35.9%) of anxiety and 52.9% (95%CI 41.20 to 64.6%) had both depression and anxiety. The highest median score was 7.4 for overall performance, followed by 7.0 for physical function and 6.3 for total function. The emotional function (2.5.1), cognitive function (1.7), and social function (0.0)and financial difficulty (3.3) .

The most common symptoms were fatigue (3.3), pain (5.0), insomnia (3.3), loss of appetite (3.3) and nausea/vomiting (1.7). In this sample, patients did not report dyspnea and diarrhea as shown in Table 1.

**Table 1:** Characteristics of socioeconomic, clinical, anxiety, depression and life quality of patients in palliative care (n = 70), Brazil, 2023

Characteristics	n (%); median (IIQ^1^)
***Age***	66.0 (58.2; 74.0)
***Sex***	
Female	42 (60.0%)
Male	28 (40.0%)
***Tumor Types***	
Aggressive tumors	41 (58.6%)
Less aggressive tumors	29 (41.4%)
***Primary cancer site***	
Colon	14 (20.0%)
Breast	9 (12.9%)
Lung	7 (10.0%)
Recto	7 (10.0%)
Stomach	5 (7.1%)
Pancreas	5 (7.1%)
Esophagus	4 (5.7%)
Unknown	3 (4.3%)
Ovary	3 (4.3%)
Prostate	3 (4.3%)
Melanoma	2 (2.9%)
Nasopharynx	2 (2.9%)
Bladder	1 (1.4%)
Larynx	1 (1.4%)
LMC	1 (1.4%)
Oropharynx	1 (1.4%)
Penis	1 (1.4%)
Rim	1 (1.4%)
***Questionnaire outcomes***	
Depression	31 (44.3%)
Anxiety	18 (25.7%)
Depression/Anxiety	37 (52.9%)
***Domains*** **EORTC-C30**	
***Overall Performance***	
Median (IIQ^1^)	7.9 (6.7, 10.0)
***Total Function***	
Median (IIQ^1^)	6.7 (5.0, 10.0)
***Physical Function***	
Median (IIQ^1^)	7.0 (4.7, 8.7)
***Cognitive Function***	
Median (IIQ^1^)	1.7 (0.0, 4.6)
***Emotional Function***	
Median (IIQ^1^)	2.5 (1.0, 4.8)
**Social role**	
Median (IIQ^1^)	0.0 (0.0, 3.3)
***Financial difficulty***	
Median (IIQ^1^)	3.3 (0.0, 6.7)
***Dyspnea***	
Median (IIQ^1^)	0.0 (0.0, 0.0)
***Pain***	
Median (IIQ^1^)	5.0 (0.0, 6.7)
***Fatigue***	
Median (IIQ^1^)	3.3 (2.2, 6.7)
***Insomnia***	
Median (IIQ^1^)	3.3 (0.0, 6.7)
***Appetite loss***	
Median (IIQ^1^)	3.3 (0.0, 9.2)
***Nausea/Vomiting***	
Median (IIQ^1^)	1.7 (0.0, 5.0)
***Constipation***	
Median (IIQ^1^)	0.0 (0.0, 3.3)
***Diarrhea***	
Median (IIQ^1^)	0.0 (0.0, 0.0)

Patients with higher physical and total function scores were less likely to be depressed. However, participants with higher emotional scores were more likely to be depressed (OR = 1,55,p < 0,001). No physical symptoms were statistically associated with depression in the univariate analysis. A lower chance of depression in patients who had a higher score in the Overall Performance (OR = 0.89), a result similar to that presented in the physical function score (OR = 0.81), total function (OR = 0.82). And a greater chance of depression in patients who showed a higher score in cognitive function (OR = 1.22; p = 0.046), emotional function (OR = 1.32), social role (OR = 1.19)and on the fatigue scale (OR = 1.20) Regarding anxiety and depression we found an association between the outcome and the following domains of the EORTC-C30 questionnaire: overall performance, physical function, total function, emotional function, in addition to the symptoms fatigue, loss of appetite, and nausea/vomiting (Table 2 ). Patients with higher scores in overall performance (OR = 0.76), physical function (OR = 0.79), and total function (OR = 0.82) showed a lower chance of anxiety and depression. However, higher scores on emotional function, fatigue (OR = 1.19), lack of appetite (OR = 1.12), and nausea/vomiting (OR = 1.21) increased the chance of the patient being classified as having anxiety and depression according to the HADS scale, as shown in Table 2 .

**Table 2:** – Factors associated with depression, unadjusted analysis, in oncological patients under palliative care at a hospital service (n = 70), Brazil, 2023

Outcome	Variables	OR^1^	95% CI^1^	p-valor
*Anxiety*				
	Tumor type			
	Aggressive tumors	1		0.800
	Less aggressive tumors	0.87	0.28, 2.57	
	**Sex**			
	Female	1		
	Male	0.68	0.21, 2.05	0.504
	**Age**	0.98	0.94, 1.03	0.515
	Domains of EORTC-C30*			
	Overall Performance	0.84	0.68, 1.03	0.101
	Physical Function	0.81	0.67, 0.98	**0.034**
	Total Function	0.81	0.69, 0.94	**0.007**
	Cognitive Function	1.18	0.96, 1.47	0.120
	Emotional Function	1.55	1.24, 2.04	**< 0.001**
	Social role	1.13	0.95, 1.35	0.158
	Financial difficulty	1.14	0.97, 1.34	0.107
	**Symptoms**			
	Dyspnea	1.06	0.88, 1.26	0.476
	Pain	1.10	0.95, 1.29	0.213
	Fatigue	1.11	0.93, 1.32	0.245
	Insomnia	1.08	0.94, 1.25	0.279
	Loss of appetite	1.12	0.99, 1.28	0.076
	Nausea/Vomiting	1.08	0.92, 1.26	0.348
	Constipation	1.07	0.93, 1.23	0.320
	Diarrhea	1.05	0.86, 1.27	0.590
***Depression***	**Tumor type**			
	More aggressive tumors	1		0.681
	Less aggressive tumors	0.82	0.31, 2.13	
	**Sex**			
	Female	1		0.844
	Male	0.91	0.34, 2.38	
	**Age**	1.00	0.96, 1.05	0.882
	Domains of EORTC-C30*			
	Overall Performance	0.80	0.64, 0.97	**0.033**
	Physical Function	0.81	0.67, 0.96	**0.022**
	Total Function	0.82	0.71, 0.94	**0.007**
	Cognitive Function	1.22	1.01, 1.51	**0.046**
	Emotional Function	1.32	1.09, 1.64	**0.007**
	Social role	1.19	1.01, 1.42	**0.049**
	Financial difficulty	1.10	0.96, 1.27	0.190
	**Symptoms**			
	Dyspnea	0.99	0.84, 1.17	0.937
	Pain	1.09	0.95, 1.25	0.223
	Fatigue	1.20	1.02, 1.43	**0.030**
	Insomnia	1.00	0.88, 1.14	0.941
	Appetite loss	1.06	0.95, 1.19	0.274
	Nausea/Vomiting	1.08	0.94, 1.26	0.273
	Constipation	1.04	0.92, 1.19	0.510
	Diarrhea	1.12	0.93, 1.36	0.244
***Depression or anxiety***	**Tumor type**			
	More aggressive tumors	1		0.259
	Less aggressive tumors	0.58	0.22, 1.50	
	**Sex**			
	Female	1		0.696
	Male	0.83	0.31, 2.16	
	**Age**	1.00	0.96, 1.04	0.954
	Domains of EORTC-C30*			
	Overall Performance	0.76	0.60, 0.94	**0.016**
	Physical Function	0.79	0.65, 0.95	**0.016**
	Total Function	0.82	0.70, 0.94	**0.008**
	Cognitive Function	1.21	1.00, 1.51	0.061
	Emotional Function	1.45	1.17, 1.87	**0.002**
	Social role	1.17	0.99, 1.42	0.078
	Financial difficulty	1.12	0.97, 1.30	0.144
	**Symptoms**			
	Dyspnea	1.00	0.85, 1.19	0.969
	Pain	1.13	0.98, 1.30	0.093
	Fatigue	1.19	1.01, 1.41	**0.043**
	Insomnia	1.04	0.92, 1.18	0.545
	Appetite loss	1.12	1.00, 1.27	**0.049**
	Nausea/Vomiting	1.21	1.04, 1.45	**0.021**
	Constipation	1.03	0.91, 1.18	0.601
	Diarrhea	1.15	0.96, 1.46	0.164

Note: OR = Odds Ratio, 95% CI = Confidence interval

After estimating the multivariate model adjusted by sex, greater physical functionality and total functionality reduced the probability of depression by 25.0% (p = 0.025) and 23.5% (p = 0.006), respectively. Patients with a high level of quality of life and functionality had a lower chance of depression, representing a 28% and 25% lower probability of this outcome compared to participants with a low level of quality of life and functionality. Patients with a high level of fatigue have a 22% greater chance of depression compared to those with a low level. .

Similar to previous results, patients with a higher level of quality of life and physical functionality and total functionality showed a lower chance of depression/anxiety compared to those with a low level of quality of life and functionality, with a reduction of 36.9, 28 0.0 and 21.9%, respectively. Conversely, participants with higher scores for loss of appetite (OR = 1.12), nausea/vomiting (OR = 1.22), and fatigue (OR = 1.21) had a higher probability of anxiety and depression, as shown in Table 3 .

**Table 3:** Factors associated with anxiety, depression, depression/anxiety, unadjusted analysis in oncological patients under palliative care at a hospital service (n = 70) Brazil, 2023

Outcome	Variables	OR^1^	95% CI^1^	p-valor
*Anxiety*	Domains of EORTC-C30*			
	Physical Function	0.80	0.65, 0.97	**0.025**
	Total Function	0.81	0.69, 0.94	**0.006**
	Domains of EORTC-C30*			
***Depression***	Overall Performance	0.78	0.62, 0.96	**0.023**
	Physical Function	0.80	0.66, 0.96	**0.019**
	Total Function	0.82	0.71, 0.94	**0.007**
	**Symptoms**			
	Fatigue	1.22	1.03, 1.46	**0.024**
***Anxiety or Depression***	Domains of EORTC-C30*			
	Overall Performance	0.73	0.57, 0.91	**0.009**
	Physical Function	0.78	0.64, 0.94	**0.013**
	Total Function	0.82	0.70, 0.94	**0.008**
	**Symptoms**			
	Fatigue	1.21	1.02, 1.45	**0.031**
	Loss of appetite	1.12	1.00, 1.27	**0.048**
	Nausea/Vomiting	1.22	1.04, 1.46	**0.019**

NOTE: OR = Odds Ratio, 95% CI = Confidence interval; Domains of EORTC-C30 = Questionnaire Domains of EORTC-30 (an increase of 10 units)

## Discussion

The present study showed a prevalence of 44.3% of depression, 28.5% of anxiety, and 52.9% of both symptoms in patients with advanced cancer undergoing palliative care at Hospital Ibiapaba’s Oncology Sector in Barbacena, Minas Gerais, Brazil. There was a lower chance of depression, anxiety, and both outcomes in patients with greater physical functionality, total functionality, and Overall Performance. Additionally, three symptoms increased the chance of depression/anxiety: nausea/vomiting, fatigue, and loss of appetite.

Anxiety and depression are common symptoms in patients with advanced cancer in palliative care who have a high burden of symptoms [5, 7–9, 11, 12]. Death anxiety manifests as dread, panic, maladaptive behavior, impaired coping mechanisms, or somatic problems [7, 9], fueling the vicious cycle between mental distress and physical symptoms [7, 9].

The prevalence of depression and anxiety presented in our study is higher than that observed by Smith et al. (2003) and Hee et al. [9, 11]. In the survey carried out in England, in Manchester, 25% of patients had anxiety and 22% depression [9], and in China, the percentage of anxiety was 29%, and depression was 11% [11]. However, it shows findings very similar to those observed by Azevedo [20] found that 42% of the 115 patients in his study had depression, and Ferreira [21] used the HADS scale and found that 31.33% of the 233 patients had probably or possible anxiety, and 26.18% had probable or possible depression.

The percentage of depression and anxiety in patients with cancer undergoing palliative care, measured by HADS, showed significant variability of results depending on the country/continent in which the study was carried out, the type of cancer studied, and the performance status of the patients [7–12]. A higher prevalence of depression was observed in developing countries [10]. A higher probability of anxiety and depression was evidenced in patients with a more significant burden of symptoms (dry mouth, pain, fatigue, lack of appetite, and lower sense of well-being) [8] and in patients with gastrointestinal and urogenital cancer compared to hematologic and head and neck cancers [7].

According to the authors, anxiety is more commonly perceived in patients with a recent cancer diagnosis, whereas depression is found in patients in the later stages of the disease, beyond therapeutic possibility [7–12, 20, 21]. The main factors associated with depression in cancer patients were a history of depression before cancer diagnosis [7, 22], younger age, advanced disease at diagnosis, and uncontrolled symptoms [23–25].

In our study, anxiety, depression, and both outcomes remained associated with the overall quality of life, and the dimensions of total function, physical function. Patients with the highest values dimensions of the total functionality and physical functionality of EORTC QLQ-C30 arrested less than depression, anxiety, and both combined results. In addition, we find less chance of depression and depression/anxiety in patients with a quality of life according to the EORTC QLQ-C30 scale. Confirming the findings of other studies [7, 8, 12], it is noteworthy that anxiety and depression were associated with global health and emotional and cognitive functioning after adjusting to the severity of the disease and pain intensity [9].

Another important result of our study was the biggest chance of depression in patients with high fatigue score (OR = 1.22), and more likely to anxiety and depression jointly in those with greater symptoms of fatigue (OR = 1.21), loss of appetite (OR = 1.12), nausea and vomiting (OR = 1.22), results similar to that observed in other studies [8, 9, 12]. Likewise, studies identified a positive correlation between depression and fatigue [9, 12]. And in the study developed by Delgado et al. [8] in his studies showed that patients with anxiety (HADSD ≥ 8), when compared to those without this symptom, had greater intensity of the loss of appetite (p = 0.005), fatigue (p = 0.001), pain (p = 0.008), worse well -being (p = 0.0007) and depression (p < 0.0001). And patients with depression (HADSD ≥ 8) also showed greater intensity of drowsiness (p = 0.017), fatigue p < 0.0001), and worse well-being compared to without depression [8].

Fatigue was the symptom most frequently observed in this study. The findings by Salvetti [14] and corroborators confirmed the findings of this study because fatigue was also the most prevalent symptom followed by insomnia, pain, and appetite loss. The same happened in the study by Silveira and collaborators [26]. Fatigue is “an unpleasant symptom that includes sensations ranging from exhaustion to tiredness, impacting the individual’s ability to perform daily activities. This symptom is present in about 75% of patients with advanced cancer and may be associated with anemia, malnutrition, neuroendocrine impairment, and muscle dysfunction, among other factors [6, 27]. Tracking this symptom in advanced cancer patients should be performed by a validated scale, associated with good clinical evaluation to identify reversible underlying causes to treat them through pharmacological and non-pharmacological measures [6, 27].

Nausea and vomiting are symptoms experienced by 68% of cancer patients at some point in their illness, and in the last six months of life, there is an increase of more than 40% in the prevalence of nausea and vomiting [6, 28]. Towards our results, studies have shown that the lack of control and relief for nausea/vomiting generates physical, cognitive, and psychosocial suffering [6, 28, 29]. Except in situations where they are contraindicated, antiemetic medications should be administered, associated with non-pharmacological interventions such as dietary nutritional follow-up, psychological services, acupuncture, and acupressure [6, 30–32].

Loss of appetite is also a prevalent symptom in advanced cancer patients [5] and has been related to worse quality of life, anxiety, and depression [8–[9, 11]–12]. Ehret et al. [33], in their article “Should Loss of Appetite Be Palliated in Patients with Advanced Cancer?” I concluded that the decision on the management of this symptom should be based on the expanded discussion among health professionals, patients, and family members, to assess how important appetite is for patients and their families. The management of this symptom will benefit from pharmacological drugs and nutritional and psychological follow-up [33].

The results of our study corroborate the relationship between mental suffering (depression and anxiety) with a higher quality of life, total functionality (Physical Function, Roller Performance, Emotional Function, Cognitive Function, and Social Function), and symptoms, indicating the need for control and Symptom relief, for the best quality of life of patients with advanced cancer in palliative care. However, they should be assessed with caution, as our data was collected during the months of the second wave of the COVID-19 pandemic in Brazil, which may have a period effect increasing the psychological suffering of patients with advanced cancer. In addition, the cross-sectional design of the study, in which the outcomes (anxiety and depression) and independent variables were collected simultaneously, does not allow for determining temporality between mood, quality of life, and symptoms (fatigue, loss of appetite, nausea/vomiting).

Despite its limitations, our study points to the need for psychological support. All patients with CP should be tracked for psychological suffering and associated factors to identify those who need additional evaluation and treatment. Despite the existence of international guidelines for managing these symptoms, these remain poorly controlled [5, 6, 11]. Confirming this statement, a study conducted in China found that 61% of advanced cancer patients with symptoms did not receive any treatment, only 10.93% received non -pharmacological intervention for symptoms, 1.77% received pain treatment with Opioids, and 1.99% received psychotropic medicines for mental suffering [11]. Concerning situations, physical symptoms affect patients’ quality of life, increase the days of hospitalization and anticipate death [5, 6, 11].

## Conclusion

This study found high prevalence of anxiety and depression in cancer patients under palliative care, and were associated with overall quality of life, overall functioning, physical functioning, and symptoms of fatigue, nausea/vomiting, and loss of appetite. Examining the patient’s functions will assist the clinician in alleviating symptoms of anxiety and depression, giving cancer patients in palliative care more dignity.

## Data Availability

The datasets generated and/or analyzed during the current study are not publicly available due Brazilian Law of Data Protection but are available from the corresponding author upon reasonable request.
